# The CA19-9 and Sialyl-TRA Antigens Define Separate Subpopulations of Pancreatic Cancer Cells

**DOI:** 10.1038/s41598-017-04164-z

**Published:** 2017-06-22

**Authors:** Daniel Barnett, Ying Liu, Katie Partyka, Ying Huang, Huiyuan Tang, Galen Hostetter, Randall E. Brand, Aatur D. Singhi, Richard R. Drake, Brian B. Haab

**Affiliations:** 1 Van Andel Research Institute, Grand Rapids, MI USA; 2 Fred Hutchinson Cancer Research Center, Seattle, WA USA; 3 University of Pittsburgh Medical Center, Pittsburgh, PA USA; 4 Medical University of South Carolina, Charleston, SC USA; 5 Michigan State University, East Lansing, MI USA

## Abstract

Molecular markers to detect subtypes of cancer cells could facilitate more effective treatment. We recently identified a carbohydrate antigen, named sTRA, that is as accurate a serological biomarker of pancreatic cancer as the cancer antigen CA19-9. We hypothesized that the cancer cells producing sTRA are a different subpopulation than those producing CA19-9. The sTRA glycan was significantly elevated in tumor tissue relative to adjacent pancreatic tissue in 3 separate tissue microarrays covering 38 patients. The morphologies of the cancer cells varied in association with glycan expression. Cells with dual staining of both markers tended to be in well-to-moderately differentiated glands with nuclear polarization, but exclusive sTRA staining was present in small clusters of cells with poor differentiation and large vacuoles, or in small and ill-defined glands. Patients with higher dual-staining of CA19-9 and sTRA had statistically longer time-to-progression after surgery. Patients with short time-to-progression (<2 years) had either low levels of the dual-stained cells or high levels of single-stained cells, and such patterns differentiated short from long time-to-progression with 90% (27/30) sensitivity and 80% (12/15) specificity. The sTRA and CA19-9 glycans define separate subpopulations of cancer cells and could together have value for classifying subtypes of pancreatic adenocarcinoma.

## Introduction

Pancreatic cancers display significant diversity in their rates of growth and dissemination and in their responses to drugs, leading to uncertainty in determining the best treatment for each patient. Methods to subclassify pancreatic cancers to predict behavior clearly would be valuable both for patient care and drug research. At the histomorphological level, several variants of pancreatic ductal adenocarcinoma (PDAC) have clinical implications^1^. For example, medullary adenocarcinomas usually are microsatellite-instable and have better prognoses^2^; adenosquamous cancers may respond better to platinum-based agents^3^; colloid cancers typically are less aggressive^4^; undifferentiated carcinomas frequently have amplifications of mutant *KRAS* and are more aggressive^5^, and undifferentiated with osteoclast-like giant cells may have better prognoses than conventional PDAC^6^. These variants, however, are the minority; most are conventional PDAC. Further subtypes of PDAC may require molecular biomarkers for identification, as they would be largely indistinguishable by morphology.

Stratification by particular DNA mutations may provide additional guidance in treatment. Mutations in DNA repair genes, such as *BRCA2* or *PALB2*, are often sensitive to poly(ADP-ribose) polymerase (PARP) inhibitors and cisplatin^7, 8^, and mutations in the mismatch repair genes confer increased susceptibility to immune checkpoint inhibitors^9^. Subtyping by genome-wide signatures also has shown promise for subtyping tumors. Recent studies identified recurrent classes that were distinguished by genes relating to development, differentiation, and immune infiltration^10–13^, and a squamous class showed shorter survival^13^. Nevertheless, further research is needed to link practical biomarkers with cancer behavior.

One of the difficulties associated with the molecular profiling of pancreatic cancers is heterogeneity in the tumors. Multiple types of cells may be present at variable levels, all amidst hugely varying backgrounds of extracellular matrix. With methods that use homogenized tissue, one cannot determine which cells produce each marker, or whether certain cells co-express various markers. A bioinformatics method could sort out the information indirectly^11^, but with limited precision. An additional challenge stems from the possibility that more than one subpopulation of cancer cell could coexist in a tumor. In support of this concept, studies involving isolations of tumor cells with particular stem-cell antigens suggest a minority subtype with heightened tumor-forming capability^14, 15^, and analyses of tumor cells in the blood suggest a subpopulation that is able to disseminate prior to clinical manifestations of a primary tumor^16, 17^. As indirect evidence, the fact that chemotherapy often reduces tumor volume without eliminating the cancer suggests a subpopulation of cancer cells that is more resistant to treatment than the rest.

To distinguish between individual cells in their expression of one or more markers, a cell-by-cell analysis is required. In the present research we took such an approach using multimarker immunofluorescence (IF)^18^. The method involves probing a single section of formalin-fixed, paraffin-embedded (FFPE) tissue with multiple rounds of multispectral immunofluorescence, each round involving two or more unique antibodies. Multimarker IF gives direct observation of the locations and morphologies of the cells producing each marker, and it is compatible with FFPE tissue, which is easier to obtain than frozen tissue.

We were particularly interested in glycan expression. In previous research we identified a glycan that is a strong serological biomarker of pancreatic cancer^19^. It performed as well as the current best serological biomarker for pancreatic cancer, CA19-9, which also detects a glycan, and it was elevated in about half of the patients with low CA19-9, indicating independent regulation. These facts led us to speculate that the glycan, which we call sTRA, is produced by a different subpopulation of cancer cells than produce the CA19-9 antigen. To test that hypothesis, we sought to immunologically detect sTRA and CA19-9 in tumor tissue and test for differences in location, morphology, and molecular expression of the cancer cells that produce each glycan. Furthermore, we asked whether particular glycan levels show an association with the rate of progression of pancreatic cancer.

## Results

### The sTRA glycan is elevated in PDAC independently from CA19-9

**Figure 1:**
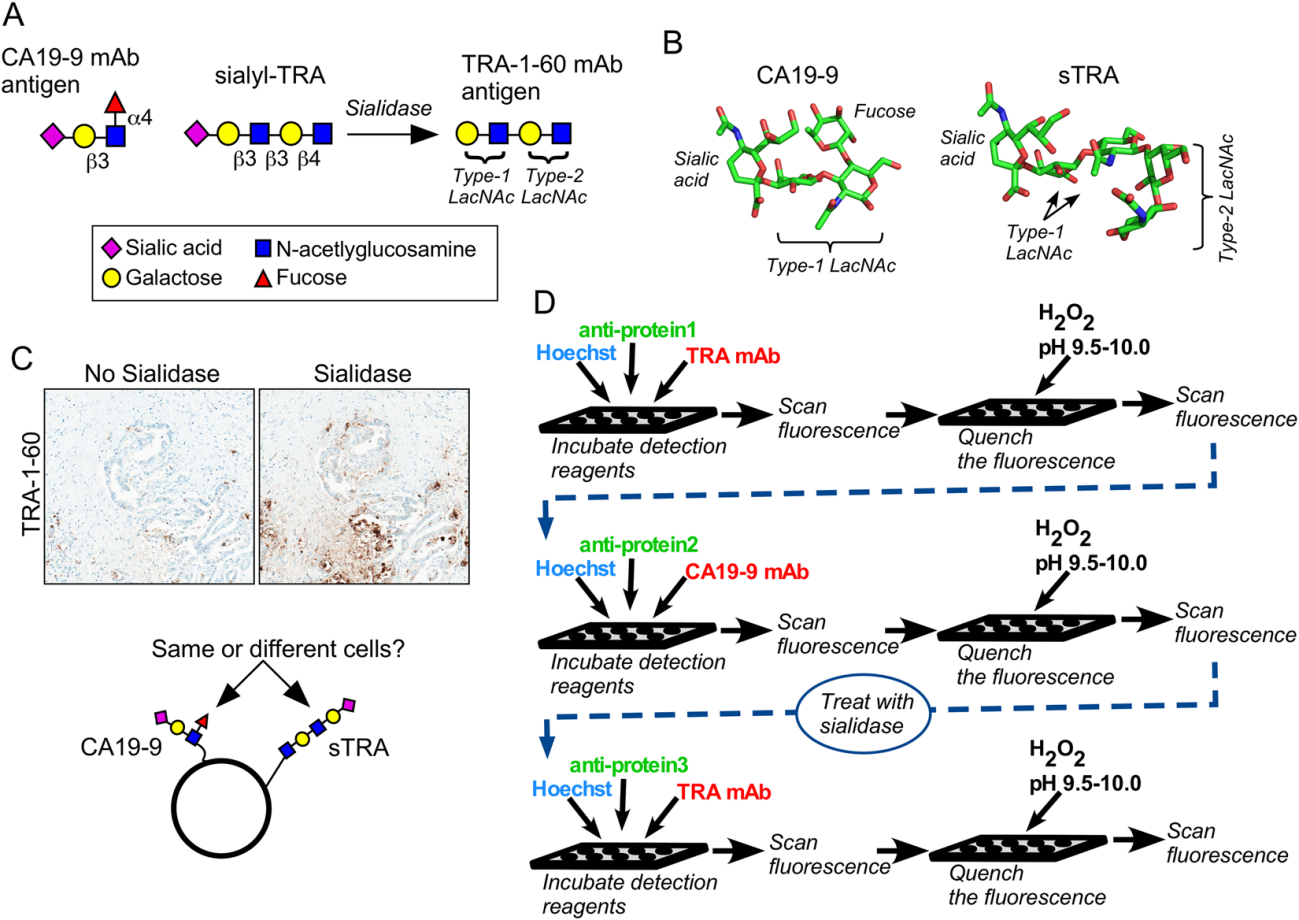
The CA19-9 and sialyl-TRA (sTRA) antigens. (**A**) In order to detect sTRA, we treat the sample with sialidase prior to applying the TRA-1-60 monoclonal antibody (mAb). (**B**) The CA19-9 and sTRA glycans have similar structures except for the presence of fucose in the CA19-9 antigen. (**C**) The treatment of pancreatic cancer tissue with sialidase leads to increased binding of the TRA-1-60 mAb, revealing the presence of sTRA in the tissue. The overlap with the CA19-9 antigen is not known. (**D**) The schematic shows the process we used for multimarker immunofluorescence. Between the second and third rounds, we treated the tissue with sialidase to remove sialic acid from sTRA, enabling detection by the TRA-1-60 mAb.

The CA19-9 antigen is a tetrasaccharide (Fig. 1A) that can be detected with high specificity using monoclonal antibodies (mAbs). The TRA-1-60 and TRA-1-81 mAbs^20^ detect a tetrasaccharide that, unlike the CA19-9 antigen, is neither fucosylated nor sialylated^21^. To indirectly detect the sialylated version of the TRA antigen (referred to as sTRA), we incubate the labeled TRA mAb to detect and mask the non-sialylated antigens, treat with sialidase, and again incubate the labeled TRA mAb (Fig. 1A) to detect the newly-exposed antigens. A view of the structures shows the similarity between the CA19-9 and sTRA antigens, as well as their main difference of a branched fucose on CA19-9 (Fig. 1B).

The treatment of tumor tissue with sialidase markedly increased staining by the TRA antibody (Fig. 1C), indicating higher levels of the sialylated antigen relative to the non-sialylated. The central question explored here is whether the cancer cells producing sTRA are different in their locations and characteristics than the cancer cells producing the CA19-9 antigen (referred to simply as CA19-9).

To probe this question in primary tissue we chose multimarker immunofluorescence, which allows for the performance of multiple antibody incubations and staining with hematoxylin and eosin (H&E) on a single section from FFPE tissue. We performed three rounds of immunofluorescence, in each round detecting blue fluorescence from a DNA stain, green fluorescence from a Cy3-labeled antibody against a protein, and red fluorescence from a Cy5-labeled protein against a glycan (Fig. 1D). We applied the TRA and CA19-9 mAbs in the first and second rounds, respectively, treated the section with sialidase, and then applied the TRA mAb again in the third round.

**Figure 2:**
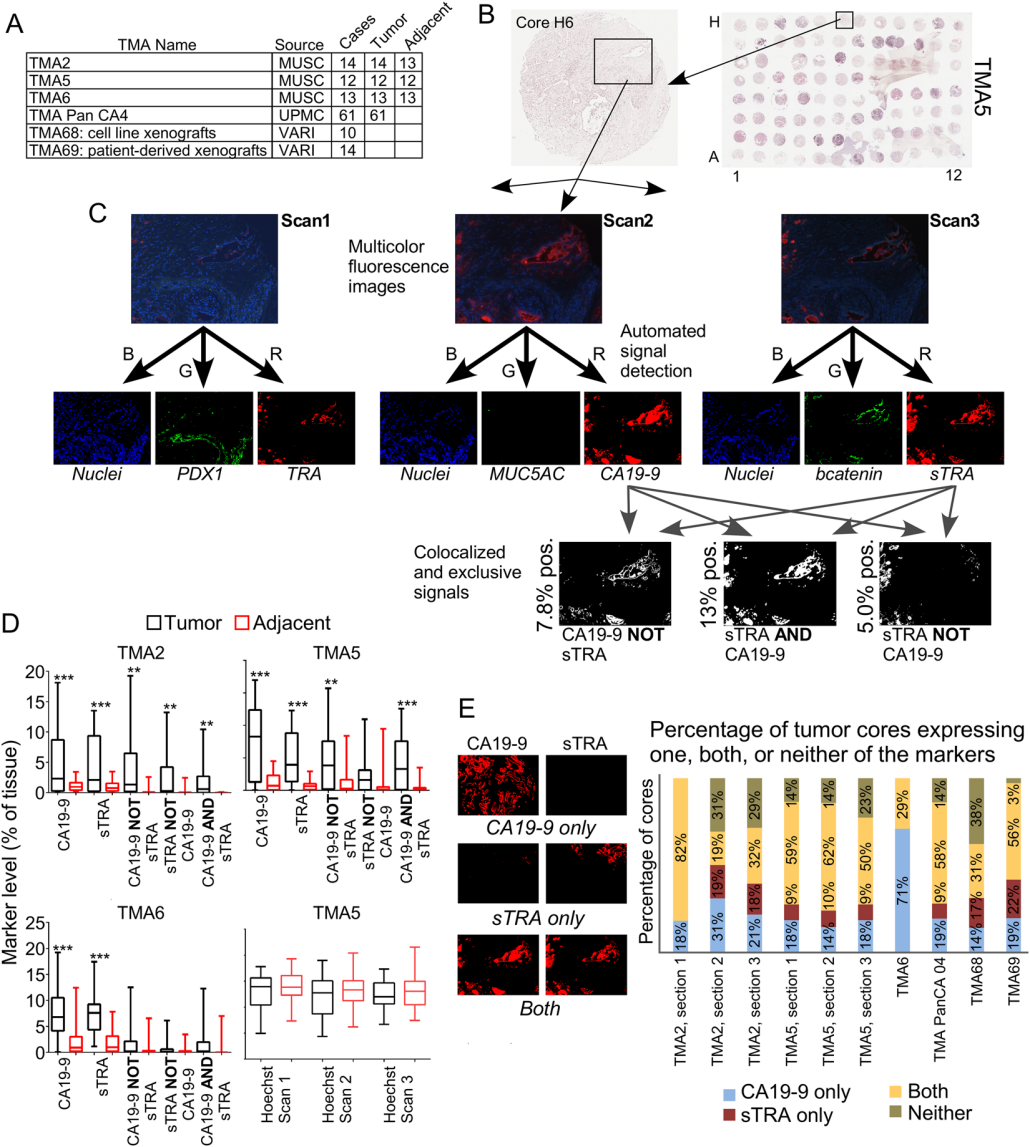
Quantifying signals in tissue microarrays. (**A**) We used six TMAs from three sources. Four TMAs contained primary tumor and adjacent tissue, and two contained tumors from xenografts. (**B**) We collected images that were tiled across the TMA. Each image captured a portion of a core, as represented by the box. (**C**) We separately analyzed the blue, green, and red channels from each of the three rounds of immunofluorescence, resulting in nine images per region. The first step was to locate the signals in each image. Next we quantified the amount of signal in each individual image as well as the amount of exclusive or colocalized signal among various combinations of images. (**D**) We compared each of the quantified signals between the tumor cores and the adjacent tissue cores. The amount of signal from the nuclei (stained by Hoechst 33258) was equivalent between tumor and adjacent tissue in all comparisons. (**E**) We quantified the occurrences of tumor tissue that contains one, both, or neither of the markers in each of the TMAs. The images provide examples of each type.

We applied the method to six separate TMAs, four made from primary tumors and two made from xenograft tumors (Fig. 2A). We acquired tiled images across the entire TMA. The field-of-view of each image was 500 × 400 μm, requiring 6–9 images to cover a core (Fig. 2B). Each field-of-view comprised a stack of 35 images collected at various wavelengths, from which we selected the three images corresponding to the fluorescent dyes used here. We quantified the amounts of signal using custom software that employs the SFT signal-finding algorithm^22^, and we then quantified the relationships between the signals from each color. Of particular interest was the possibility that the exclusive expression of a particular marker, i.e. the presence of one marker in the absence of another, could be a marker of phenotype. We therefore designed software to quantify exclusive expression as well as colocalization (Fig. 2B).

We first wanted to know which glycans or glycan combinations are elevated in pancreatic tumors relative to adjacent tissue from the pancreas. We examined the signals from CA19-9, sTRA, CA19-9 in the absence of sTRA (referred to as CA19-9-only), sTRA in the absence of CA19-9 (referred to as sTRA-only), and colocalized expression of both CA19-9 and sTRA (referred to as dual-labeling). Both CA19-9 and sTRA were significantly elevated in the tumors, as were the exclusive and dual expression of the markers in most cases (Fig. 2D). Using combined data from TMAs 2, 5, and 6, each of the five markers was significantly elevated (p < 0.001 based on Wilcoxon signed rank test, with false discovery rate <0.001 accounting for the five markers tested) in the tumors relative to paired adjacent tissue (Table S1). Without sialidase pretreatment, detection with the TRA-1-60 mAb did not show significant elevations in the tumors (not shown).

We asked whether the sTRA expression occasionally occurs in locations and tumors that are separate from CA19-9, or whether it is simply an overlapping subset of CA19-9 expression. To begin, we recorded how often a tumor core had only CA19-9 expression, only sTRA expression, both, or neither (Fig. 2E). (If the marker was expressed in >1% of the tissue pixels, we counted the core as expressing the marker.) We saw the repeatable occurrence of tumors with predominant sTRA or CA19-9, with good agreement across the TMAs and among individual sections of the same TMA (Fig. 2C). An aberration was the first section of TMA2, which was the first section run and had some poor quality images due to lack of optimization. Also, TMA6 had no cores with only sTRA expression, which we attributed to natural variation between tumors because the images showed no obvious defects in data quality. The two TMAs containing xenograft tumors were similar to the other TMAs, indicating that the expression of each marker persists in culture and in animals. The consistent occurrence of tumors that predominantly express sTRA (Fig. 2E), as well as the elevation of sTRA-only regions in the tumors relative to adjacent tissue (Fig. 2D), affirm that sTRA is a marker of pancreatic cancer independent of CA19-9.

### The sTRA and CA19-9 glycans identify spatially and morphologically distinct subsets of cancer cells

**Figure 3:**
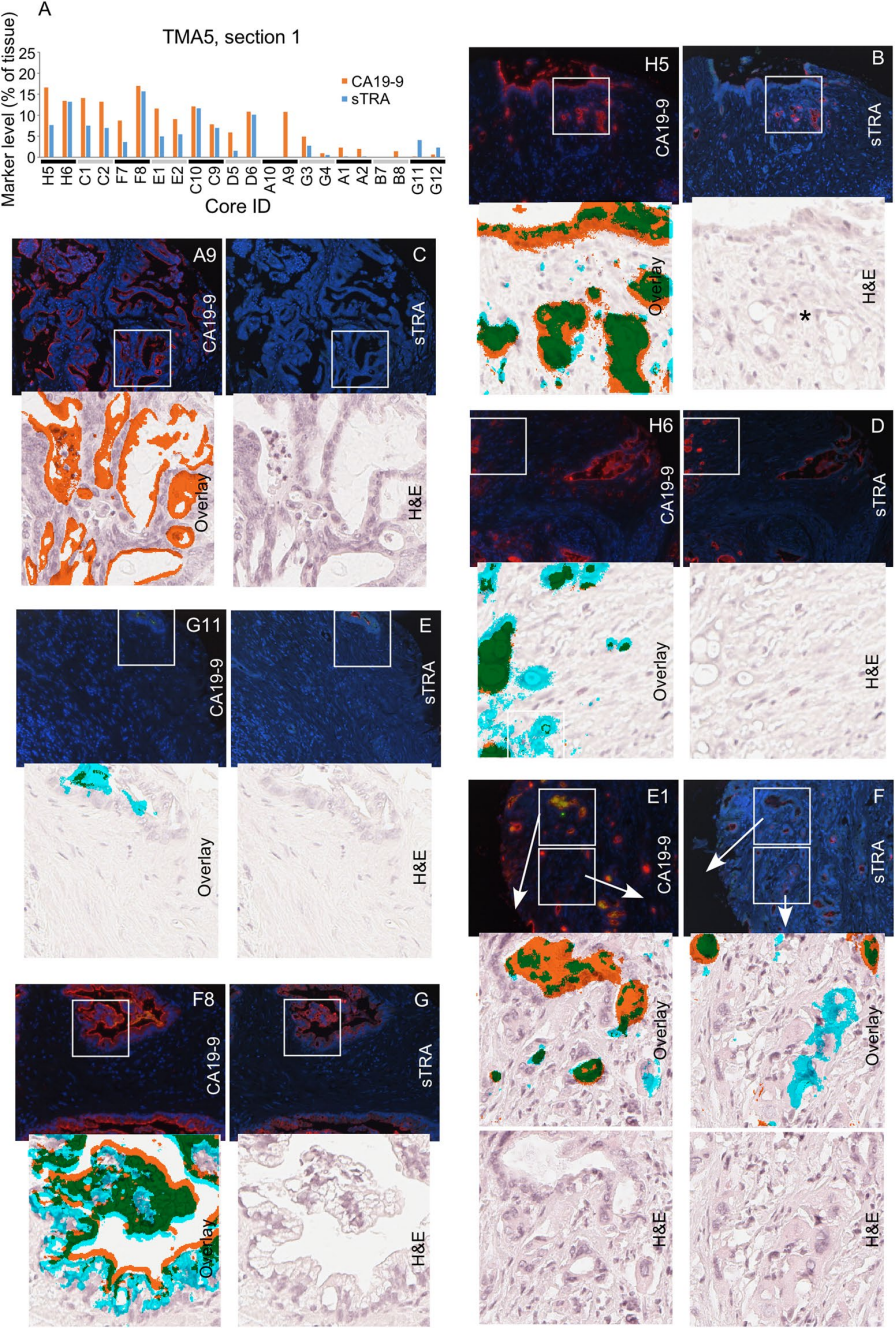
Cellular morphologies associated with each glycan. (**A**) The cores had various levels of each antigen. The TMA contained two cores from each tumor, presented in pairs in the column graph. (**B**–**G**) The top two images in each group are the raw fluorescence from the second (left) and third (right) rounds of immunofluorescence. The red signal in the left image is CA19-9, and the red signal in the right image is sTRA. The lower images in each group are zoomed pictures corresponding to the white box. The lower left image is the H&E image overlaid with the detected signals. The signal from CA19-9 is orange, the signal from sTRA is cyan, and the overlapping signal is green. The lower right image is from the H&E stain. The asterisk in panel B marks vacuolated, invasive cells.

We next explored whether the cells expressing one or the other marker have divergent locations or histomorphologies. The quantification of TMA5 showed that cores were present with various levels of each marker (Fig. 3A). Some consistent patterns emerged upon examination of the tissue. In areas of well-differentiated PDAC, CA19-9 staining generally was more prevalent than sTRA (Fig. 3B). High CA19-9 in the absence of sTRA also occurred in moderately-to-poorly differentiated PDAC (Figs 3C and S1). Cells that primarily or exclusively expressed sTRA showed other morphologies. A common feature was vacuolated cells^23^ (Fig. 3B and D); and less common was sparse, moderately-differentiated glands amidst heavy desmoplasia (Fig. 3E). In some cases, the non-invasive ductal epithelium stained mostly with CA19-9 while the invasive cells were strongly positive for sTRA (Fig. 3B). Certain tumors showed a subpopulation of sTRA-expressing cells with large cytoplasm (Fig. 3F) adjacent to moderately-differentiated glands expressing CA19-9 (Fig. 3F). We found that the well-differentiated epithelium with foamy cytoplasm^24^ always expressed both sTRA and CA19-9 (Figs 3G and S1).

The two markers, therefore, are present in non-identical subsets of cancer cells. The cancer cells variously express either one, both, or neither of the markers, and the morphologies of the cells group into a few categories in association with the exclusive or dual expression of CA19-9 and sTRA.

**Figure 4:**
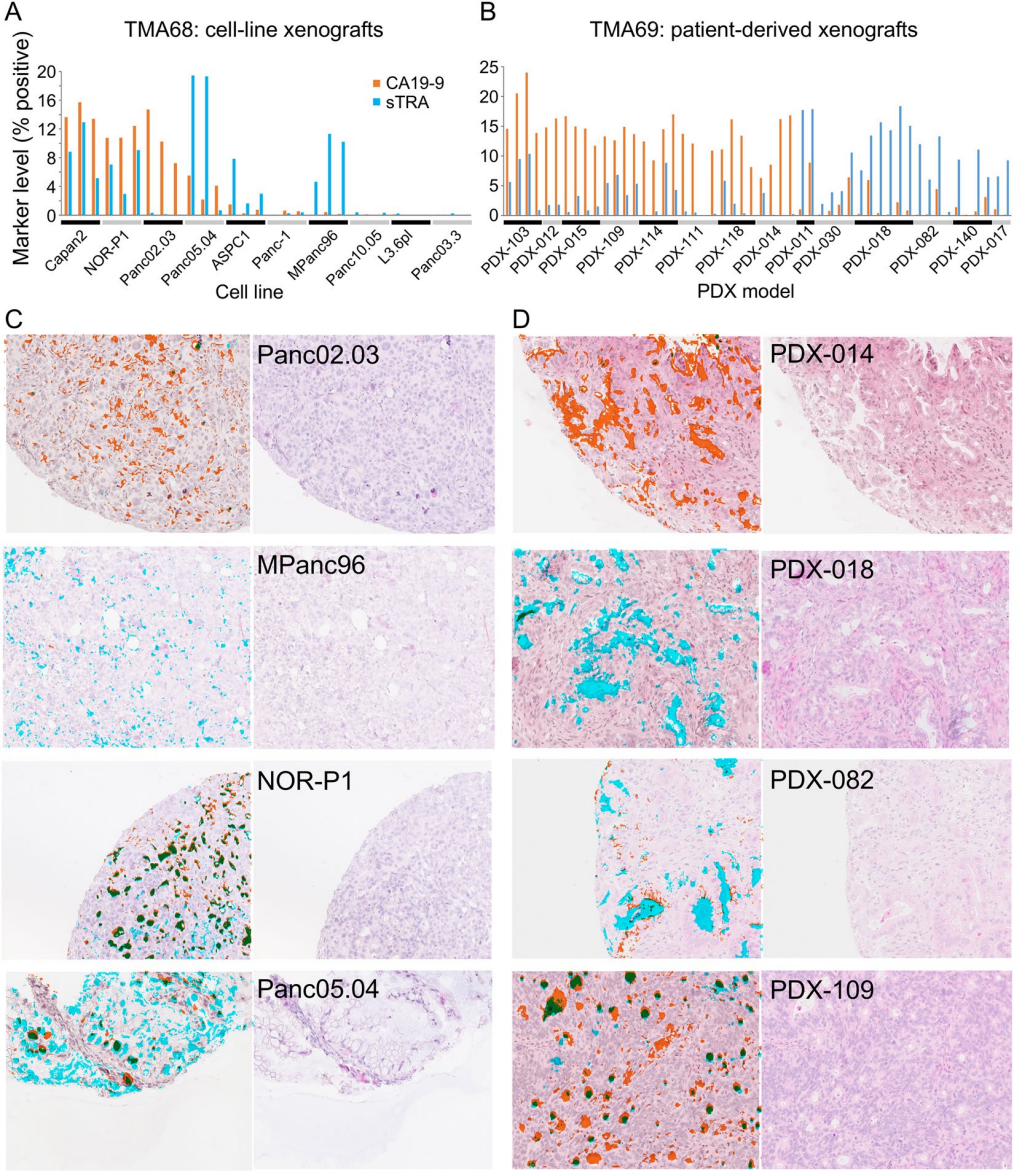
Staining and morphologies in xenografts. (**A**) The cell-line xenograft cores showed various amounts of each antigen. The three cores from each cell line are presented in groups. (**B**) Each PDX xenograft expressed either primarily CA19-9 or primarily sTRA. Each model had 2–6 cores on the TMA. (**C**,**D**) Selected images from the cell-line (**C**) and PDX (**D**) xenografts are presented. In the overlaid images at the left of each pair, CA19-9 is orange, sTRA is cyan, and the overlapping signal is green.

We asked if the above observations hold true in model systems. The results from a TMA containing cell-line xenografts and a TMA containing patient-derived xenografts were similar to the results from the primary tumors. Among the 10 cell lines on TMA68, some expressed both markers, others only one marker, and others neither (Fig. 4A). The 14 PDX models on TMA69 showed divergent expression of the two markers; eight primarily expressed CA19-9, and six primarily sTRA (Fig. 4B). The tumors from the cell-line xenografts generally showed less stroma and ductal epithelium than primary tumors, whereas the PDX models had better recapitulation of the primary tumors. The tumors expressing primarily CA19-9 or sTRA were largely similar to each other in histomorphology (Figs 4C, D, S2 and S3), although one of the cell lines with exclusive sTRA expression, Panc05.04, showed a lipid-rich phenotype (Fig. 4C). We observed this phenotype also in primary tissue with heavy sTRA staining (Fig. S1). These analyses show that the CA19-9 and sTRA expression phenotypes persist in cultured cancer cells and are not just unique to the primary tumors.

### Protein expression differs based on glycan type and differentiation

We next asked if the expression of key markers of phenotype are different between the groups identified above. We stained for various protein markers in a separate color from the glycans (Fig. 1D), acquiring measurements of three proteins in each of two sections from TMAs 2 and 5. In one section we stained for E-cadherin, vimentin, and cytokeratin 19 (CK19). We found that nearly all cells expressing either CA19-9 or sTRA also expressed CK19 and E-cadherin, regardless of morphology (Fig. S4), and that none expressed vimentin (not shown), indicating a consistent epithelial phenotype.

**Figure 5:**
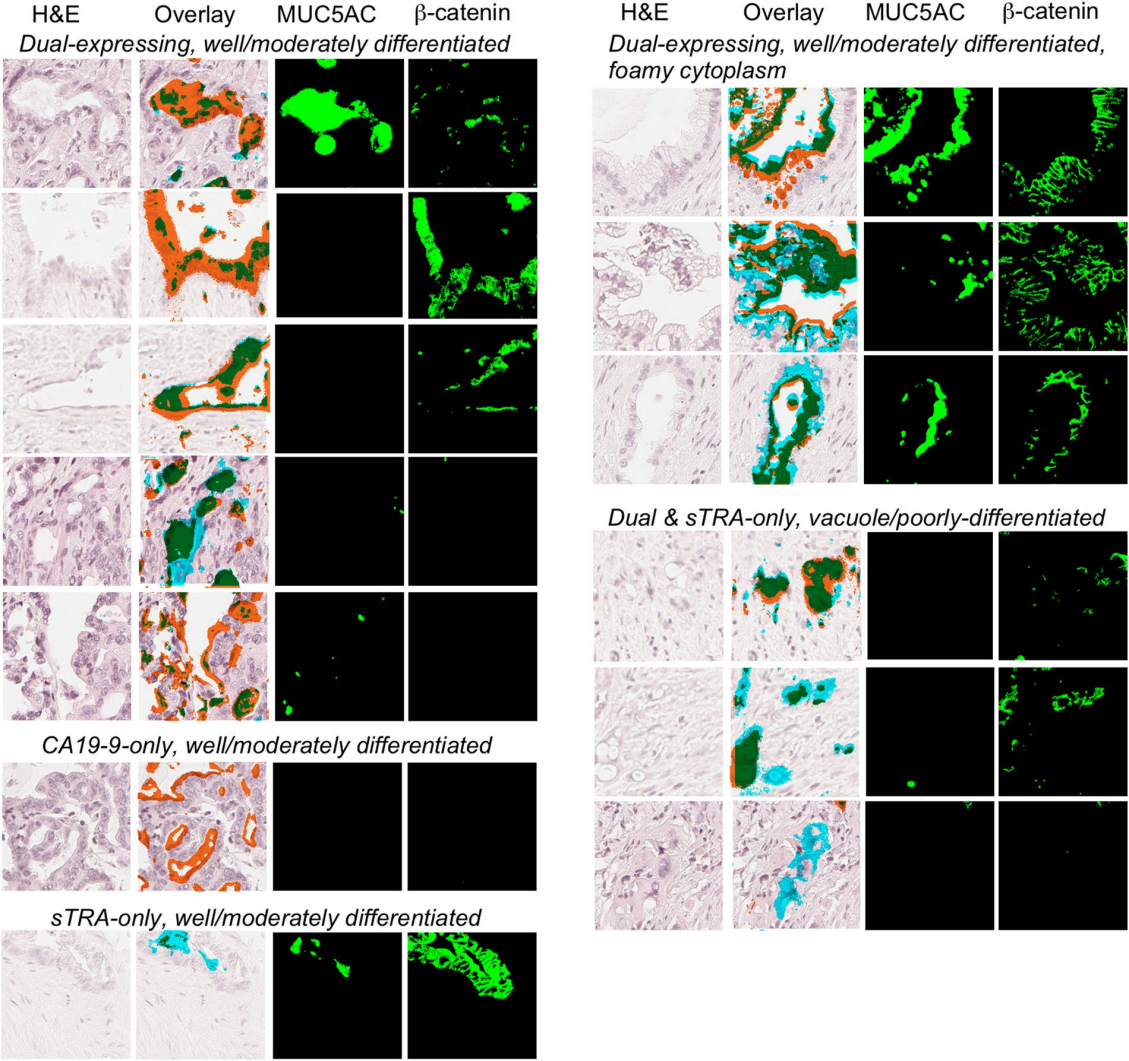
Protein expression in various cell types. The images are grouped according to glycan expression and morphological phenotype. For each region, we present the H&E image, the H&E with overlaid signal (using the color scheme in Figs 3 and 4), the signal detected for MUC5AC, and the signal detected for β-catenin.

We saw more diversity between cells in MUC5AC, a marker of neoplastic and mucin-producing glands, and β-catenin, a marker of regeneration or adhesion (Fig. 5). We probed for these proteins along with PDX-1 in another section. The well-differentiated PDACs expressing both sTRA and CA19-9 showed membranous expression of β-catenin, but the poorly-differentiated cells generally showed weaker membranous staining, consistent with a loss of epithelial adhesion. The dual-labeled epithelium with clear cytoplasm always expressed MUC5AC, as did well-differentiated neoplastic glands with luminal secretions, but the poorly-differentiated neoplastic cells never expressed MUC5AC. A tumor with exclusive expression of CA19-9 in moderately-differentiated PDAC with no surrounding stroma expressed neither β-catenin nor MUC5AC, but a moderately-differentiated PDAC secreting primarily sTRA expressed both. These analyses provide evidence that the dual-labeled cells often represent cohesive and secretory epithelia, and that the single-labeled cells are more frequently dyshesive and non-secreting, although additional phenotypes occur.

### The expression patterns of sTRA and CA19-9 predict time-to-progression

**Figure 6:**
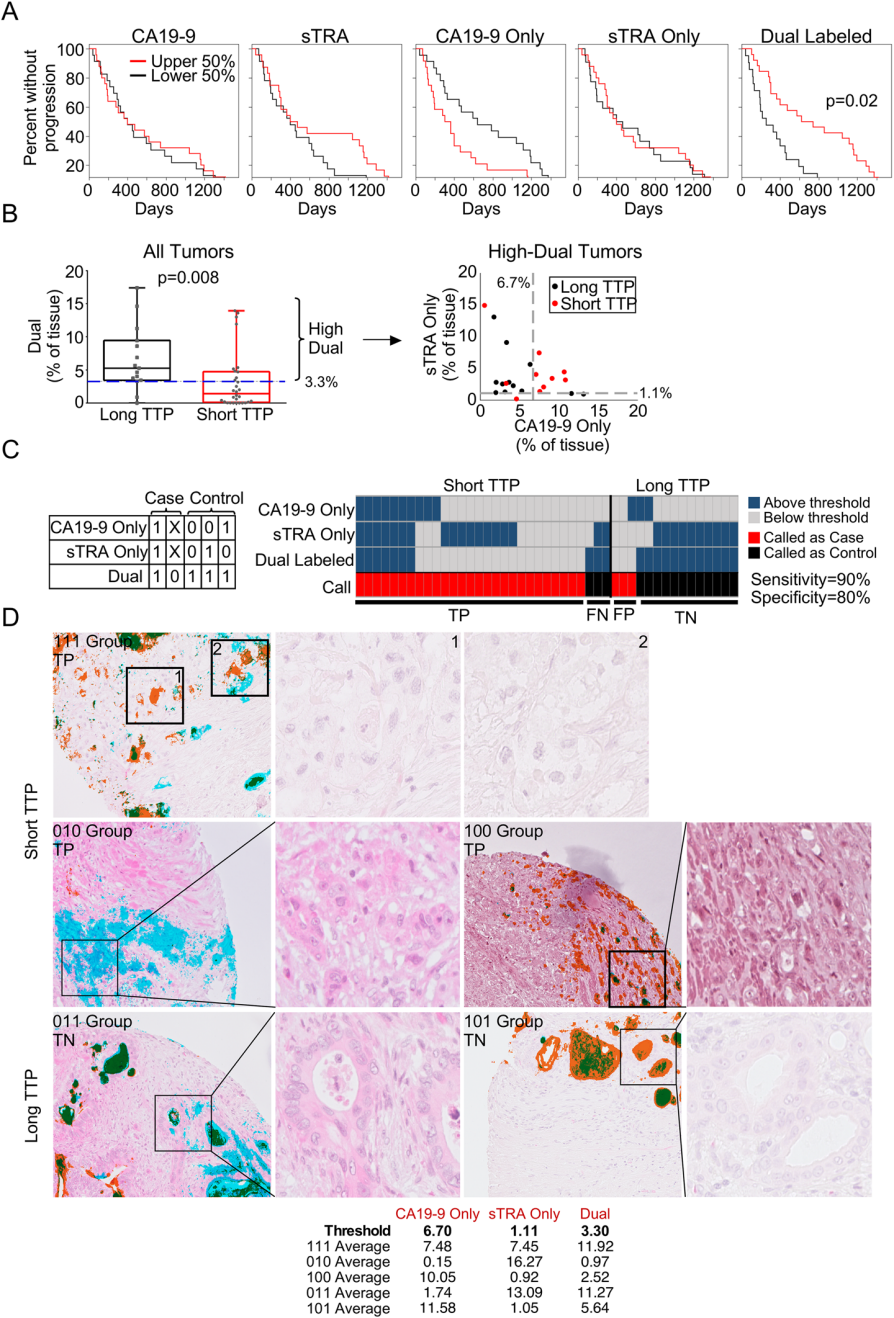
Associations between glycan type and time-to-progression (TTP). (**A**) We divided the patients into the upper and lower halves of expression of each of the five indicated marker types, and then separately plotted Kaplan-Maier curves for each. (**B**) The tumors with long TTP (>2 years) had significantly higher (p = 0.008, Wilcoxon rank sum test) dual-marker expression than the patients with short TTP. Among just the tumors with high dual-marker expression, only tumors with short TTP had high levels of both the sTRA-only and CA19-9-only markers. The dashed lines indicate the threshold for each marker, and the value of the threshold is given. (**C**) Patients that were high in all three of the indicated markers, or that were low in the dual marker, were called as cases (short TTP) and all other patients were called as controls (long TTP). The table shows the rules for the calls. A ‘1’ indicates the marker is above its threshold, ‘0’ indicates below threshold, and ‘X’ indicates either above or below. TP, true positive; FN, false negative; FP, false positive; TN, true negative. (**D**) We present H&E images both with and without overlaid signal. The colors for the overlays are the same as in Figs 3–5. A frequent observation among short-TTP patients with high levels of all three markers (top row) or low levels of the dual markers (middle row) was scattered groups of poorly-differentiated cells labeled either with sTRA or CA19-9. A common feature among long-TTP patients (bottom row) was moderately- or well-differentiated PDACs without scattered groups of single-labeled cells. The table provide the averages over all regions for each of the tumors in panel (D).

We next explored whether certain patterns of glycan expression are associated with the rate of disease progression. For one of the TMAs, Pan CA4, we had information for 45 of the 61 patients about the time from surgery to progression of the disease. We constructed Kaplan-Meier curves of time-to-progression (TTP) grouped by low (<median) and high (>=median) expression of each of the five markers (Fig. 6A). The amount of staining of neither the individual CA19-9 or sTRA markers nor their exclusive expression markers were related to TTP, but the amount of dual expression was related to TTP (p = 0.008 based on log-rank test). The expression of this glycan type was significantly different (p = 0.01 based on Wilcoxon rank-sum test) between patients with long TTP (>2 years) and those with short TTP (<2 years) (Fig. 6B).

We used the Marker State Space (MSS) method^25^ to test if any patterns of glycan expression distinguish tumors with long TTP from those with short TTP. The method revealed that below a certain threshold in dual-marker expression, most patients (20/23) had short TTP (Fig. 6B). Among the patients above the threshold in dual-marker expression, high levels of both CA19-9-only and sTRA-only were present only in patients with short TTP (Fig. 6B).

A three-marker panel consisting of CA19-9-only, sTRA-only, and dual-labeled formed a candidate biomarker for predicting TTP. By classifying patients with high expression of all three markers or with low expression of the dual marker as short TTP, we observed 90% sensitivity (27 correct out of 30) and 80% specificity (12 correct out of 15) for predicting short TTP (Fig. 6C). When we ran 10-fold cross validation five separate times, the average accuracy of panels from the training sets applied to set-aside samples was 78% (Table 1), which is less than the 87% accuracy (39/45 correct) found for the true grouping, as expected, but robust. Random groupings of the 45 samples as cases and controls resulted in no marker panels (except for one occurrence out of 50) that met the minimum performance of 80% sensitivity and 80% specificity in the training sets (Table 1). These results—the good performance in the true grouping and the poor performance in the random groupings—support the interpretation that the marker panel is detecting a true difference between short and long TTP.

**Table 1:** Results from 10-fold cross validation.

	Split1	Split2	Split3	Split4	Split5	Split6	Split7	Split8	Split9	Split10	Median
Round 1	100%	84%	73%	37%	75%	70%	50%	75%	62%	23%	71%
Round 2	53%	88%	42%	80%	53%	100%	55%	88%	88%	80%	80%
Round 3	83%	49%	62%	71%	50%	100%	53%	100%	75%	100%	73%
Round 4	100%	60%	48%	80%	75%	80%	88%	100%	50%	93%	80%
Round 5	80%	96%	100%	35%	71%	68%	88%	100%	92%	70%	84%
Random 1	—	—	—	—	—	—	—	—	—	—	—
Random 2	80%	—	—	—	—	—	—	—	—	—	—
Random 3	—	—	—	—	—	—	—	—	—	—	—
Random 4	—	—	—	—	—	—	—	—	—	—	—
Random 5	—	—	—	—	—	—	—	—	—	—	—

We used the sTRA-only, CA19-9-only, and dual-labeled tissue markers to seek panels that distinguish 30 short-TTP samples from 15 long-TTP samples (Fig. 6). Each value is the average accuracy of the panels from the training set applied to the set-aside test samples for each split (see Materials and Methods for details). The average of the medians from the five independent rounds was 78%. For the rounds marked ‘Random’, we randomly assigned a case or control status to each of the 45 samples and repeated the process. Except for Split1 of the second round, the program did not find any panels that met the minimum performance in the training sets.

An examination of the staining patterns and histomorphology also suggested differences between the groups (Figs 6D and S5). The short TTP tumors were of two types: high in all three markers, or low in the dual marker (Fig. 6C). The short-TTP tumors that are high in all three markers frequently showed clusters of poorly-differentiated cells expressing either sTRA or CA19-9 and a lack of differentiated glands (Fig. 6D, top row), and in other cases, abundant vacuolated cells or densely-populated, well-differentiated glands (Fig. S5). The short-TTP tumors that are low in the dual marker did not have differentiated glands but rather scattered cells expressing one or the other glycan (Fig. 6D, middle row). The long-TTP tumors with expression of the dual marker generally showed well-differentiated PDACs with polarized nuclei and sometimes with vacuole-type cytoplasm (Fig. 6D, bottom row). The three short-TTP tumors that were misclassified did not have well-differentiated glands, nor did the misclassified long-TTP tumors (Fig. S6); additional modifications to the biomarker panel may be required for such tumors.

We asked whether the glycan levels associated with location and type of recurrence (distant or local) or presence of SMAD4 staining (the absence of which is a surrogate for SMAD4 genetic deletion). We did not see significant associations among the 12 patients for which we had the information, but we saw suggestive trends, such as higher dual-marker expression in patients with local recurrence, and higher CA19-9-only staining in lesions that are negative for SMAD4 (Table S2).

The protein expression in the cancer cells was similar to the protein expression presented above, but with higher β-catenin (not shown). Differences in protein levels and morphologies may have been induced by the prior treatment with chemotherapy of the tumors in TMA Pan CA4. In aggregate, these analyses provide preliminary indications that the quantification of the exclusive and dual expression of sTRA and CA19-9 could be useful for classifying tumors and for predicting the risk of disease progression.

## Discussion

Here we show that subpopulations of cancer cells in pancreatic adenocarcinomas are distinguishable by whether they express sTRA, CA19-9, or both. Tumors variously displayed one or more of the subpopulations, sometimes more than one in the same tumor. Each subpopulation had its own characteristics of morphology. Cells expressing both markers typically were part of well-differentiated and mucin-secreting PDACs, whereas those expressing just one were often poorly differentiated and vacuolated and never mucin secreting. Evidence that the glycan-defined subpopulations have predictive value comes from the associations with TTP. We found indications that tumors with short TTP come in at least two varieties: one with an absence of dual-labeled cells, and another with high levels of both the CA19-9-only cells and the sTRA-only cells. Nearly all patients with long TTP had high levels of the dual-labeled cells without high levels of the single-labeled cells. The findings suggest that the dual-labeled glands indicate a functioning and recovering pancreas, while the dispersed, single-labeled cells mark uncontrolled growth and dispersion. The predictive value of the biomarkers must be validated in follow-up research; at this point, the current research confirms that the sTRA and CA19-9 glycans in combination identify separate subpopulations of cancer cells. In the second place, the research provides an initial look at differences between the subpopulations.

An enabling component of the present work was the automated and quantitative analysis of multimarker immunofluorescence data. The huge number of images generated in this study would have been impossible to analyze manually, and the analysis would have been only semi-quantitative. The method used here allowed quantification of image data from multiple markers and multiple TMAs and ultimately identification of staining patterns that showed associations with outcome. Another important component was the quantification of exclusive and dual expression of the markers, which provided better classifications than individual measurements. We foresee such a system having usefulness for research and eventually for clinical applications. In clinical applications, automated image analysis could help to remove inter-operator variability or to pick out rare or subtle features. For example, in OGCs, where the malignant type can be a histiocyte-like sarcomatoid carcinoma cell^6^, automated image analysis could find signals from a stain for such cells amidst an overwhelming background of non-malignant cells.

Most of the previous studies aimed at categorizing pancreatic tumors concentrated on protein or genomics markers. An integrated analysis of mutational status, expression profiles, and histopathology found four subtypes of tumors, defined respectively as low exocrine and high squamous differentiation; increased pancreas-specific progenitor programs; high exocrine and/or endocrine features; or increased expression of immune-specific genes^12^. Other research emphasized the epithelial-mesenchymal transition (EMT) as a means of identifying invasive cells (see reviews refs 26, 27). The biomarkers of EMT were not highly specific to cancer cells, but immunostaining for SMAD4—a key node in signal transduction relating to EMT and other functions—may be useful for predicting distant metastasis^28^. Further research focused on developmental pathways, stem-cell antigens, markers of acinar-ductal metaplasia, among others^29–31^.

Linking the glycan types found here to the biological programs and genomics classes mentioned above could be important for further defining and understanding the subpopulations. We could not directly link our data to the genomics classes, but some inferences are possible. For example, the squamous subtype found earlier had low pancreatic differentiation and worse prognosis, suggesting a connection to the single-labeled sTRA and CA19-9 cells in the present study, which were often in nests of undifferentiated cells with no glandular differentiation and were associated with short TTP. Secondly, the previously-defined progenitor subtype had high pancreatic differentiation and mucin expression, suggesting a link with the dual-labeled, mucin-secreting cells found here. Such comparisons could facilitate studies of the biology of tumors, but for practical application, distilling the information down to a small number of immunostains may have significant value. The use of a small number of markers limits the possible number of subgroups; cellular stains provide a direct look at minority cancer cells within complex backgrounds; and cell-surface markers open the opportunity for immunological targeting.

Studies of glycans in subpopulations of cancer cells are less common than studies of proteins and nucleic acids, but they have strong foundations. Several glycans are widely used as markers of cell type, including the CD15 antigen for neutrophils^32^, the TRA-1-60 and TRA-1-81 antigens for induced pluripotent stem cells^33^, the ABO antigens for red blood cells, and the target of the *Lycopersicon esculentum* lectin for endothelial cells^34^. Considering that specific glycans frequently have roles in regulating cellular interactions, it follows that the glycans would be remodeled when cells change states. Pancreatic cancer cells exhibit such behavior. Glycans altered in pancreatic cancer include CA19-9^35^, members of the Lewis blood group family^36^, and ABO blood group antigens^36^. Except for CA19-9, such glycans were not highly specific to cancer cells. In contrast, sTRA appears to be highly specific to cancer cells, at least in the context of pancreatic tumors. A similar glycan that is present on a glycolipid called sialosyllactotetraosylceramide, or LSTa, has been observed in small-cell lung carcinoma^37^ and glioma^38^, and its non-sialylated version is a marker of embryonic stem cells and induced pluripotent stem cells^33^. Consistent with expression on newly-differentiating cells, the sTRA antigen is potentially the precursor of the CA19-9 antigen^39^. These factors suggest that a stem-like population expresses the non-sialylated TRA antigen, and that as cells transform into PDACs, they modify the TRA antigen with sialylation and/or fucosylation. The glycans do not have fully characterized functions, but alterations by fucosylation or sialylation can modulate binding with selectins^40^ and galectins^41^, which in turn could alter signal transduction, cell differentiation, and cell migration. The functions may depend on the protein or lipid carriers of the glycans, which have not been characterized for sTRA.

A model arising from this work is that particular glycan and protein combinations define unique differentiation states of cancer cells, making it possible to differentiate tumors based on their content of each type of cancer cell. Such markers could be readily applied to cytologic smears from FNA, which can be difficult to interpret by morphology. An area for potential application would be to help determine which patients should have surgery and which should immediately begin drug treatment. If further research shows that distinct subpopulations have differential responses to available therapies, another application is to select therapeutic regimens. Considering the increasing range of drugs available for PDAC, the opportunities are expanding for matching subtypes to their optimal drugs.

## Materials and Methods

### Tissue samples and tissue microarrays

The study was conducted under protocols approved by the Institutional Review Boards at the Van Andel Research Institute, the University of Pittsburgh Medical Center, and the Medical University of South Carolina. All subjects provided written, informed consent, and all methods were performed in accordance with the relevant guidelines and regulations. The tissue samples were collected from extra portions of surgical resections for pancreatic cancer. At each site, tissue microarrays were generated from 1 mm cores of formalin-fixed, paraffin-embedded (FFPE) tissue.

### Multimarker immunofluorescence and chemical staining

We performed immunofluorescence and chemical stains on 5 μm thick sections cut from formalin-fixed, paraffin-embedded blocks. We removed paraffin, performed antigen retrieval by incubating the slides in citrate buffer at 100 °C for 20 minutes, and blocked the slides in 1X phosphate-buffered saline containing 0.05% Tween-20 (PBST0.05) and 3% bovine serum albumin (BSA) for 1 hour at RT. We labeled two primary antibodies respectively with Sulfo-Cyanine5 NHS ester (13320, Lumiprobe) and Sulfo-Cyanine3 NHS ester (11320, Lumiprobe) according to the supplier protocol. Each round of immunofluorescence used two different antibodies, one against a glycan and one against a protein (see Table S3 for details about the antibodies). After dialysis to remove unreacted dye, we prepared a solution containing both antibodies at 10 μg/mL in PBST0.05 with 3% BSA. We incubated the antibody solution on a tissue section overnight at 4 °C in a humidified chamber.

The next day, we decanted the antibody solution and washed the slide twice for 3 minutes each in PBST0.05% and once for 3 minutes in 1X PBS. We dried the slide by blotting and incubated Hoechst 33258 (1:1000 dilution in 1X PBS) for 10 minutes at RT to stain nuclei. Following two five-minute washes in 1X PBS, we added a coverslip and scanned the slide using a scanning-fluorescence microscope (Vectra, PerkinElmer). The microscope collected 35 images at each field-of-view, each image at a different emission wavelength.

We stored the slides in a humidified chamber between rounds of immunofluorescence. Prior to the next round, we removed the coverslip by immersing the slide in deionized water at 37 °C for 30–60 minutes, or until the coverslip came off, and quenched the fluorescence using 6% H_2_O_2_ in 250 mM sodium bicarbonate (pH 9.5–10) twice for 20 min. each at RT. The subsequent incubations and scanning steps were as described above.

To treat the slide with sialidase, we incubated a 1:200 dilution (from a 50,000 U/mL stock) of the enzyme (α2–3,6,8 Neuraminidase, P0720L, New England Biolabs) in 1X enzyme buffer (5 mM CaCl_2_, 50 mM pH 5.5 sodium acetate) overnight at 37 °C. We washed the slides as above prior to the following antibody incubations. The hematoxylin and eosin (H&E) staining followed a standard protocol.

### Image and data processing

We used in-house software called SignalFinder to locate pixels containing signal in each image. The program uses our recently-published SFT algorithm^22^ without user intervention or adjustment of settings. From the 35 images captured for each region, we selected the three that corresponded to the emission maxima of Hoechst 33258, Cy3, and Cy5. For each image, SignalFinder creates a map of the locations of pixels containing signal and computes the percentage of tissue-containing pixels that have signal. To arrive at a final number for each core, we averaged over all images for a core. To quantify exclusive or colocalized signals between markers, we used in-house software called ColocFinder. The program allows the user to build up expressions of AND, OR, and NOT between scans, and then quantifies the percentage of pixels that fulfill the expression. The AND operator requires signal pixels to be present in both scans, the OR operator requires pixels to be present in either scan, and the NOT operator requires pixels to be present in the first but not the second scan.

We further analyzed and prepared the data using Microsoft Office Excel and GraphPad Pro, and we prepared the figures using Canvas 14 and Canvas Draw (ACD Systems). The SignalFinder and ColocFinder programs are available upon request.

### Statistical analysis

We used Wilcoxon signed rank test to compare the distribution of a biomarker score between paired samples (e.g. tumor tissue versus adjacent tissue). In the presence of multiple biomarkers (CA19-9, CA19-9-only, sTRA, sTRA-only, Dual), false discovery rate was computed using the Benjamini & Hochberg method. We used Wilcoxon rank sum test to compare the distribution of a biomarker score between two independent groups (i.e. patients with short TTP and patients with long TTP). Kaplan-Meier curves were plotted to characterize the distribution of time-to-progression. Log-rank test was conducted to compare TTP distributions between two groups (i.e. patients with low and high marker values).

### Biomarker panel selection using MSS

We selected marker panels using the Marker State Space (MSS) method^25^. The program searches for marker “states,” or patterns of high and low marker values, that are predominant either in cases or controls and that form accurate classification rules. MSS limits the initial size of panels to 3 markers, with the option of adding markers iteratively. The MSS software is available upon request.

The MSS software has the option of 10-fold cross validation. The program randomly divides all samples into 10 groups, sets aside one group as a test set, and runs the marker search process on the remaining samples. It scans through all threshold combinations to find any panels that meet a minimum accuracy (set by the user, here 80% sensitivity and 80% specificity) in the training samples. The program then applies each panel to the set-aside samples to classify each sample as a case or a control. It compares the true states of the set-aside samples to the classifications made by the panel to determine the accuracy of the classifications. The accuracy is given as the percentage of classifications that were correct. If no panels give the minimum performance in the training set of a particular split, no panels are applied to the set-aside samples, and no accuracies are given. The program repeats the entire process for all 10 splits.

For further testing, we randomly assigned each of the 45 samples as a case or control. That is, instead of using the actual case or control status of each sample as the input to the program, we used a random assignment of case or control status for each sample. If the marker selection method were simply overfitting classifiers to the data, it would find good classifiers regardless of how the samples are grouped. But if real differences exist between the actual groups, the performance of the panels should greatly decrease when the grouping is randomized.

### Patient-derived xenograft (PDX) and cell-line xenograft models

All animal studies were approved by the VARI Institutional Animal Care and Use Committee (IACUC), and all experiments were performed in accordance with relevant guidelines and regulations. The xenograft studies used 6–8 week old mice from the VARI breeding colony.

The tissue for the PDX models was obtained from surgical resections for pancreatic cancer performed at regional hospitals in Grand Rapids, Michigan, under protocols approved by institutional review boards at the respective institutions. Unused portions of the resections selected by the attending pathologist were placed in a sterile receptacle and transported immediately on ice to the VARI. Upon receipt, the tumor tissue for implantation was placed into a sterile dish containing sterile phosphate buffered saline and carefully teased into ≤3 millimeters (longest axis) tumor fragments.

The original PDX models were developed in athymic nu/nu mice as reported earlier^42^. Dependent on tumor tissue availability, tumor fragments were implanted in a maximum of five mice. Mice for each PDX model were gender matched to the donor patient. Following administration of general anaesthesia (isoflurane), the right flank was cleaned with 70% ethyl alcohol, a small incision made, and a subcutaneous pocket created by blunt dissection. The tumor fragment was inserted into the pocket and the incision closed using a surgical staple. Immediately following surgery, the mouse received a single dose of the analgesic Ketoprofen (5 mg/kg body weight). Mice were monitored for health and tumor growth for the duration of the study, and body weights were recorded weekly. Tumorgraft volumes (½ × length × depth × height) were measured 1x/week when volumes ≤50 mm^3^ and 3x/week at tumor volume >50 mm^3^. A tumorgraft model that failed to develop within 6 months in the 1st generation mice was discontinued and the mice euthanized. When a tumorgraft reached a volume of ≥1500 mm^3^ the mouse was euthanized, and the tumorgraft was aseptically harvested.

For the PDX tumors used for this study, cryopreserved PDX fragments were thawed rapidly, rinsed in sterile phosphate buffered saline containing 1% penicillin/streptomycin (Invitrogen), and implanted into NSG mice. Following administration of general anesthesia (isoflurane), the right flank was cleaned with 70% ethyl alcohol, a small incision made, and a subcutaneous pocket created by blunt dissection. The tumor fragment was inserted into the pocket and the incision closed using a surgical staple. Immediately following surgery, the mouse received a single dose of the analgesic Ketoprofen (5 mg/kg body weight). The monitoring and harvesting were as described above. A portion of the tumor was fixed and processed in a standard manner for histological analysis and TMA construction.

To develop cell-line xenograft models, athymic nu/nu mice were injected with 1 × 10^6^ cells/100 μL phosphate buffered saline in their right flank using a 1 cc syringe with a 27 g needle. The cell lines were obtained from the American Type Culture Collection (Manassas, VA) and grown in recommended conditions prior to subcutaneous injection. The rest of the methods were identical to those described for the patient-derived xenograft models.

## Electronic supplementary material

Supplementary Information
