# Supplementary material for: The CA19-9 and Sialyl-TRA Antigens Define Separate Subpopulations of Pancreatic Cancer Cells
Source: Sci Rep. 2017 Jun 22;7:4020. doi: 10.1038/s41598-017-04164-z (PMC5481434; doi:10.1038/s41598-017-04164-z)
Supplement: Supplementary file 1 — Supplementary Information [file 41598_2017_4164_MOESM1_ESM.pdf]

Supplementary Information

The CA19-9 and Sialyl-TRA Antigens Define Separate Subpopulations of Pancreatic Cancer Cells

Daniel Barnett,[1]* Ying Liu,[1]* Katie Partyka,[1] Ying Huang,[2] Huiyuan Tang,[1] Galen Hostetter,[1] Randall E. Brand,[3] Aatur D. Singhi,[3] Richard R. Drake,[4] and Brian B. Haab[1]

[1]Van Andel Research Institute, Grand Rapids, MI
[2]Fred Hutchinson Cancer Research Center, Seattle, WA
[3]University of Pittsburgh Medical Center, Pittsburgh, PA
[4]Medical University of South Carolina, Charleston, SC

Contents

Supplementary Tables:

- Table S1. Comparison of glycan expression between tumor and adjacent tissue
- Table S2. Relationship between glycan levels and type of recurrence or SMAD4 status.
- Table S3. Antibody details

Supplementary Figures:

- Figure S1. Additional images from the primary tumors
- Figure S2. Additional images from the cell-line xenografts
- Figure S3. Additional images from the PDX xenografts
- Figure S4. E-cadherin and CK19 expression
- Figure S5. Additional images from each tumor group associated with TTP
- Figure S6. Images from tumors with misclassified TTP

Tables

Table S1. Comparison of biomarker values (averaged over two cores) between tumor and adjacent tissues. The table presents the median and interquantile range for each biomarker (averaged over two cores) for tumor tissue and adjacent tissue separately, as well as median and interquantile range for their difference in biomarker score. A significant difference between tumor and adjacent tissues was found for each biomarker.

Biomarker	Adjacent Tissue		Tumor Tissue		Difference		Tumor vs. Adjacent	
	Median	IQR	Median	IQR*	Median	IQR	p-value#	FDR$
CA19-9	0.438	(0.171,1.006)	5.769	(1.481,11.021)	3.588	(0.574,9.939)	2.22e-08	1.11e-07
CA19-9-only	0.385	(0.085,0.915)	6.089	(1.684,8.069)	4.51	(1.362,7.512)	1.3e-07	3.26e-07
sTRA	0.023	(0.01,0.201)	0.462	(0.026,6.261)	0.246	(-0.005,5.449)	0.00096	0.00096
sTRA-only	0.022	(0.003,0.216)	0.432	(0.021,3.16)	0.3	(0.004,2.547)	0.000764	0.000955
Dual	0.012	(0.001,0.033)	0.762	(0.095,9.546)	0.377	(0.078,10.429)	1.17e-06	1.96e-06

*Interquantile range
#p-value based on the Wilcoxon signed rank test
$False discovery rate

Table S2. Relationship between glycan levels and type of recurrence or SMAD4 status. The right 3 columns give the average values of % tissue pixels for each glycan. The p value was calculated using the Wilcoxon Rank Sum test. SMAD4 information was missing for 3 of 12 patients, and recurrence information was missing for 1 of 12 patients.

	N	Long TTP (%)	Short TTP (%)	CA19-9 Only	sTRA Only	Dual
**SMAD 4 loss (+)**	3	2 (22%)	1 (11%)	1.2	4.0	5.8
**SMAD 4 loss (-)**	6	1 (11%)	5 (56%)	5.7	1.4	3.2
**p value**				0.26	0.71	0.90
**Recurrence: distant**	8	2 (18%)	6 (55%)	4.3	1.3	1.2
**Recurrence: local**	3	1 (9%)	2 (18%)	3.8	4.5	10.1
**p value**				0.92	0.13	0.28

Table S3. Antibody details.

Name	Clone ID	Target	Source	Cat. no.	Species	Class	ID
Anti-Sialyl Lewis A (CA19-9)	9L426	Sialyl Lewis A	USBio	C0075-03A	mouse	IgG	1295
TRA-1-60	TRA-1-60	Terminal N-acetyl-lactosamine, type 1	Novus Biologicals	NB100-730	mouse	IgM	1497
Anti-MUC5AC	45M1	MUC5AC	ThermoScientific	MS-145-P1ABX	mouse	IgG1	1480
Anti-beta-catenin	polyclonal	Beta-catenin	R&D Systems	AF1329	goat	IgG	1578
Anti-E-cadherin	3F4	E-cadherin	Sigma Aldrich	WH0000999M1	mouse	IgG1k	1581
Anti-CK19	RCK108	Cytokeratin 19	ThermoScientific	MA1-06329	mouse	IgG1	1591
Anti-vimentin	V9	Vimentin	Sigma Aldrich	V6389	mouse	IgG1	1582
Anti-PDX1	267712	PDX1	R&D Systems	MAB2419	mouse	IgG2B	1551

Figures

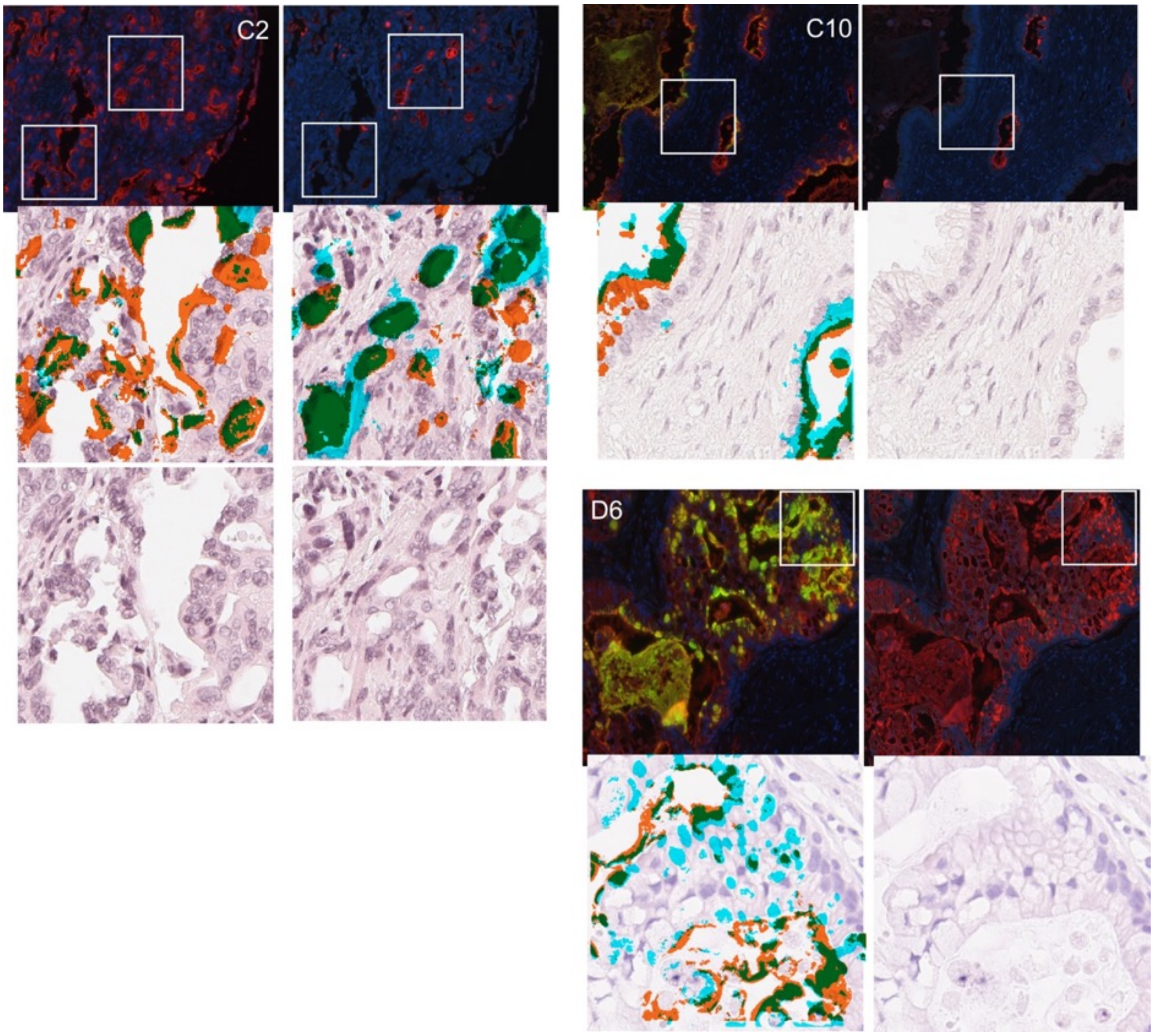

Figure S1. Additional images from the primary tumors. Cores from TMA5 are shown. The multicolor, fluorescence images are at the top of each group, with scan 2 on the left and scan 3 on the right. In scan 2, red was CA19-9 and green was MUC5AC, and in scan 3, red was sTRA and green was β-catenin. Below are H&E images from the area defined by the white box in the fluorescence image. In the overlaid H&Es, orange is CA19-9, cyan is sTRA, and green is the overlap.

Core C2 shows a moderately-differentiated duct with loose organization that stains mostly with CA19-9 (left), and small glands that secrete dual-labeled material into the lumen (right). Core C10 shows well-differentiated ducts with foamy cytoplasm that generally are labeled with both markers. Core D6 shows lipid-rich and vacuolated cells that label only with sTRA, and clusters that are dual labeled.

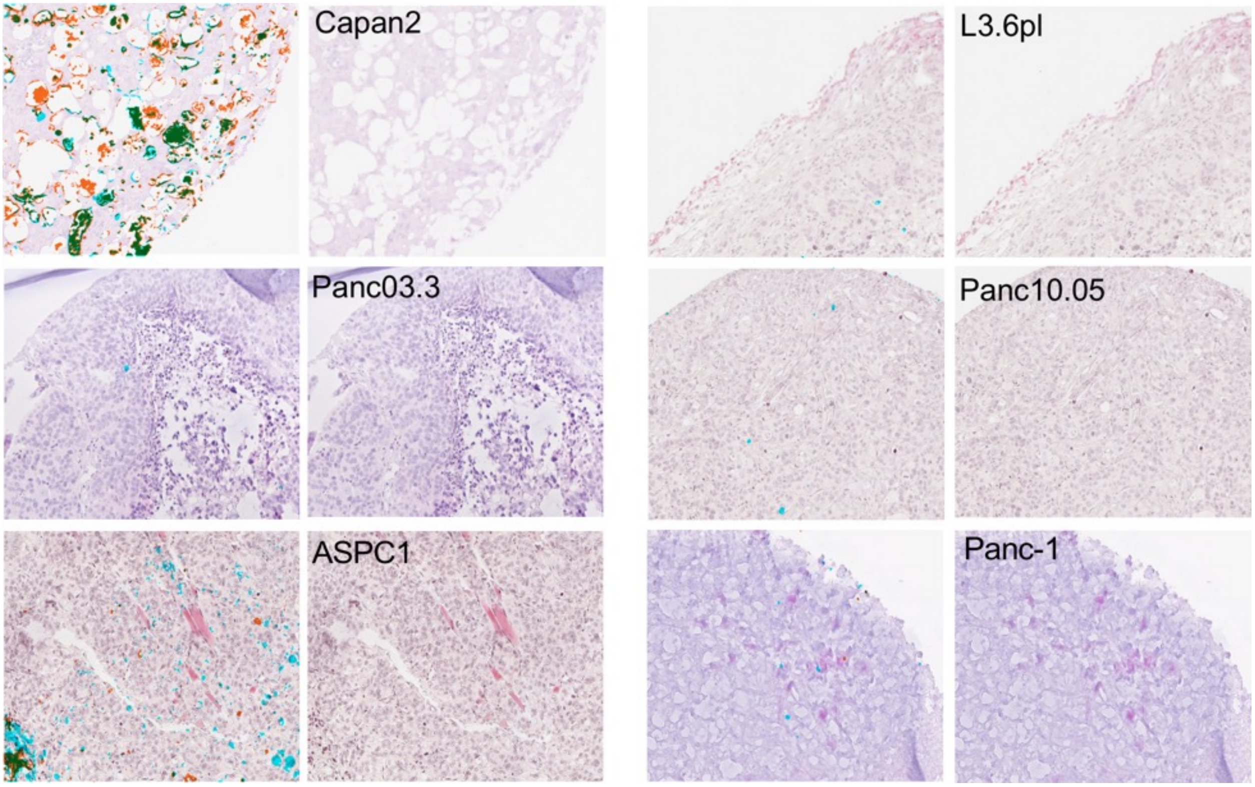

Figure S2. Additional images from the cell-line xenografts. In each pair, the H&E image is on the right and the overlaid image on the left, using the same color scheme as in Figure S1. Capan2 had high staining for both markers, ASPC1 had clear sTRA staining with little CA19-9 staining, and the rest were low in both. The L3.6pl cell line is from adenosquamous carcinoma, not ductal adenocarcinoma like the rest.

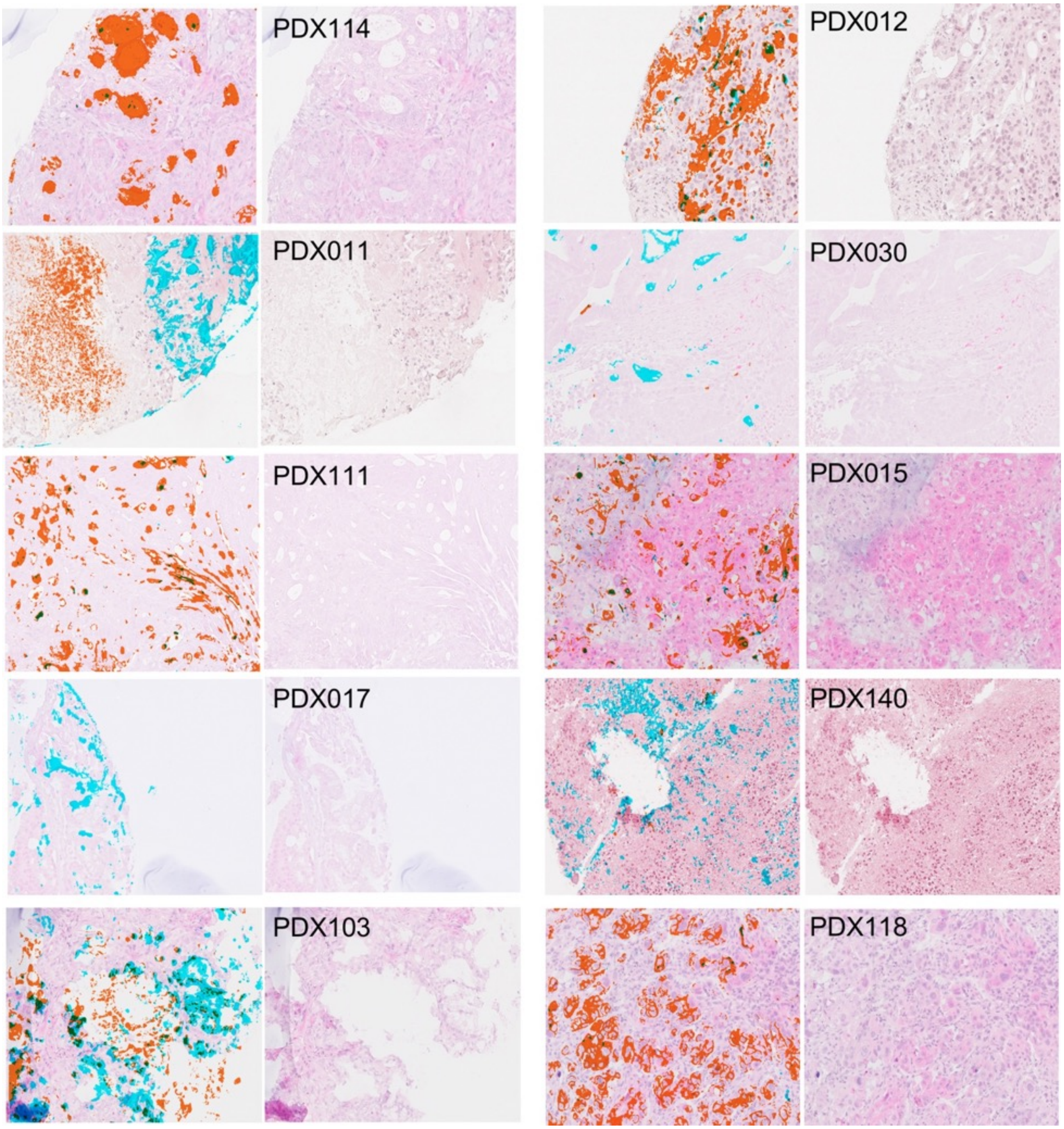

Figure S3. Additional images from the PDX xenografts. The color scheme of the overlaid images is the same as in the previous figures. Most xenograft models stain primarily with either one or the other marker, and none was wholly absent of staining.

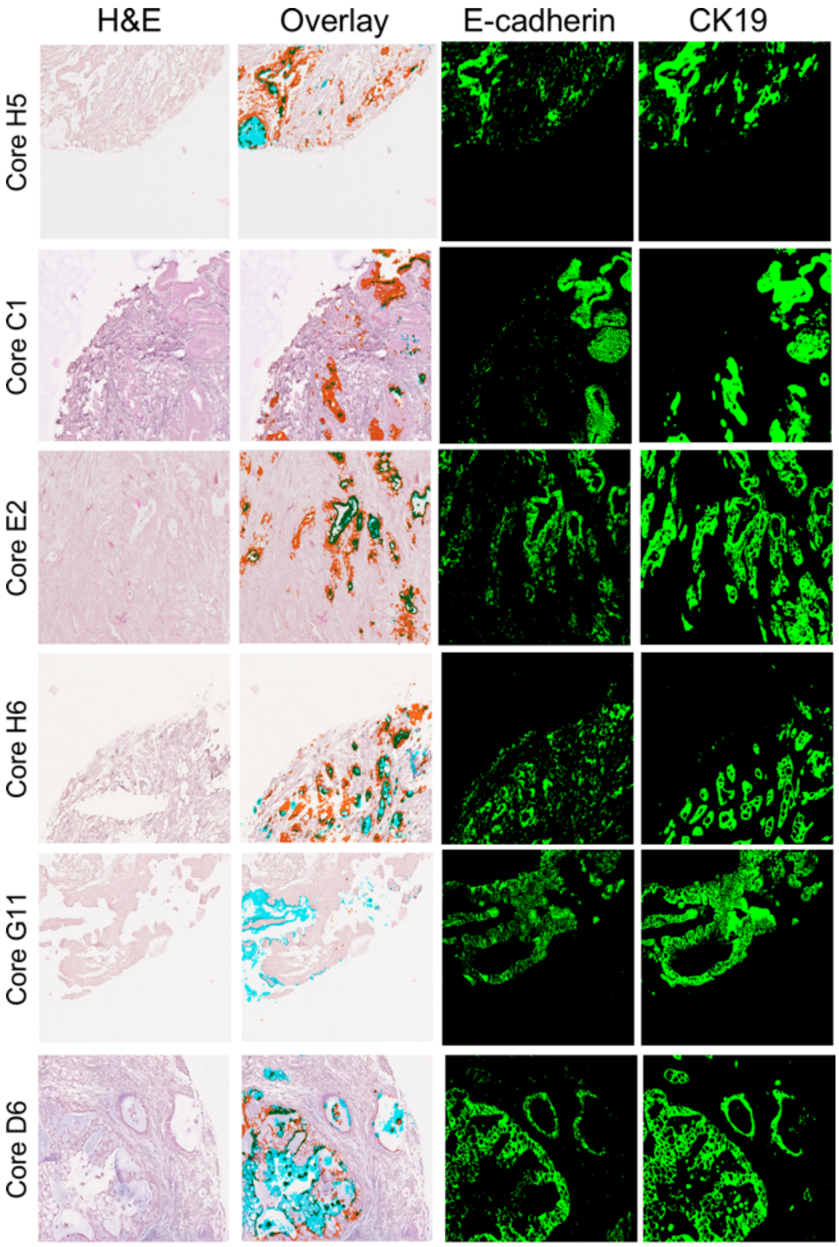

Figure S4. E-cadherin and CK19 expression. The colors for the overlaid H&E are the same as in the previous figures. The right two images show the detected signals corresponding to E-cadherin and CK19. The cells that stained for either sTRA or CA19-9 expressed E-cadherin and CK19, regardless of morphology.

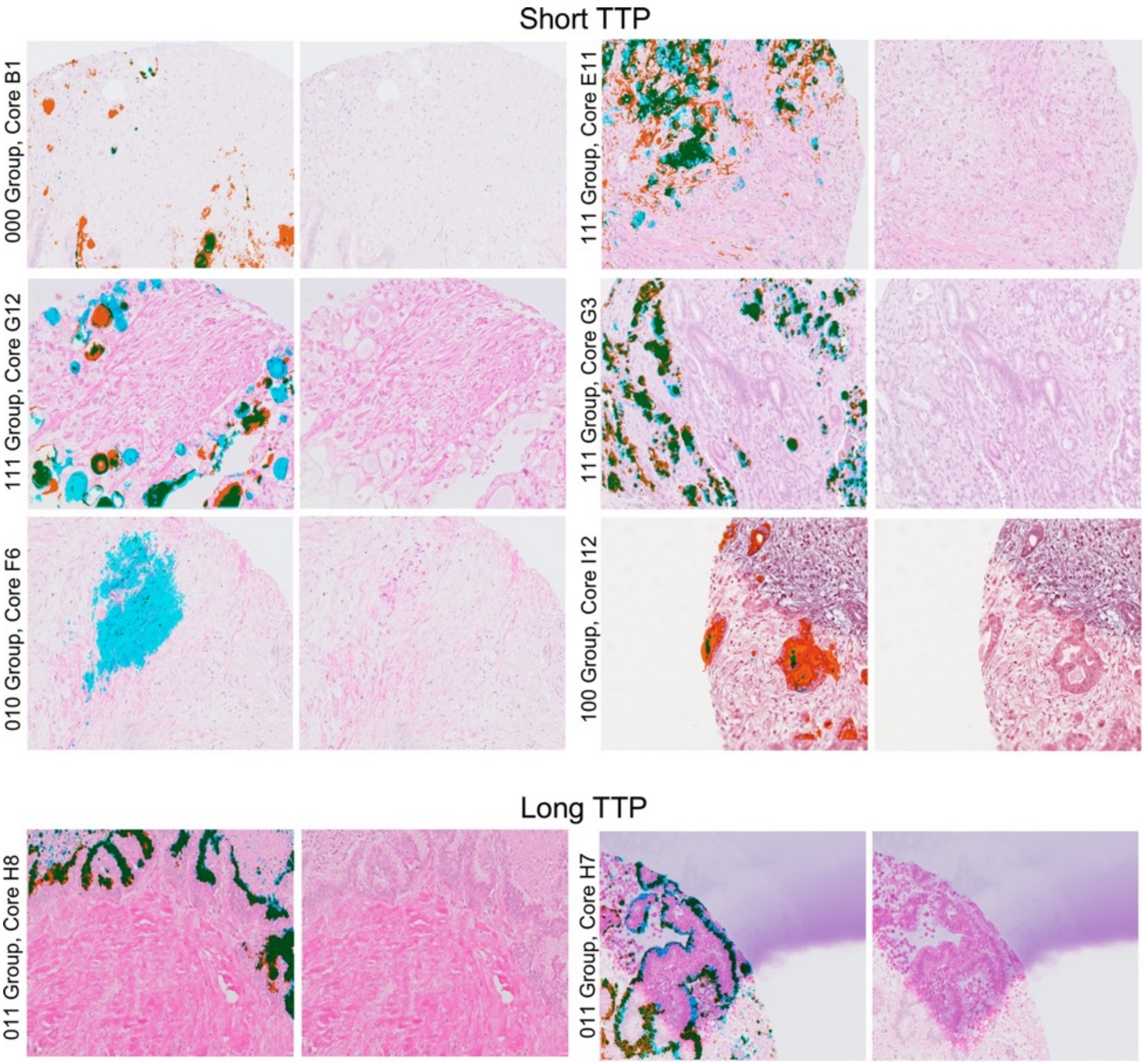

Figure S5. Additional images from each tumor group associated with time-to-progression (TTP). The color scheme in the overlaid images is the same as in previous figures. The labels on the left give the Core ID and the Group ID according to the table in Figure 6C of the main text. The Group ID gives the status of each of the three markers, where a '1' indicates above threshold, and a '0' indicates below threshold. The first number is CA19-9-only, the second is sTRA-only and the third is dual. Thus 000 indicates low in all three markers, 111 indicates high in all three, etc.

The short-TTP tumors are either high in all three markers (cores G12, E11, and G3) or low in the dual-labeled marker (cores B1, F6, and I12). The long-TTP tumors are high in the dual-labeled marker but not in all three of the markers.

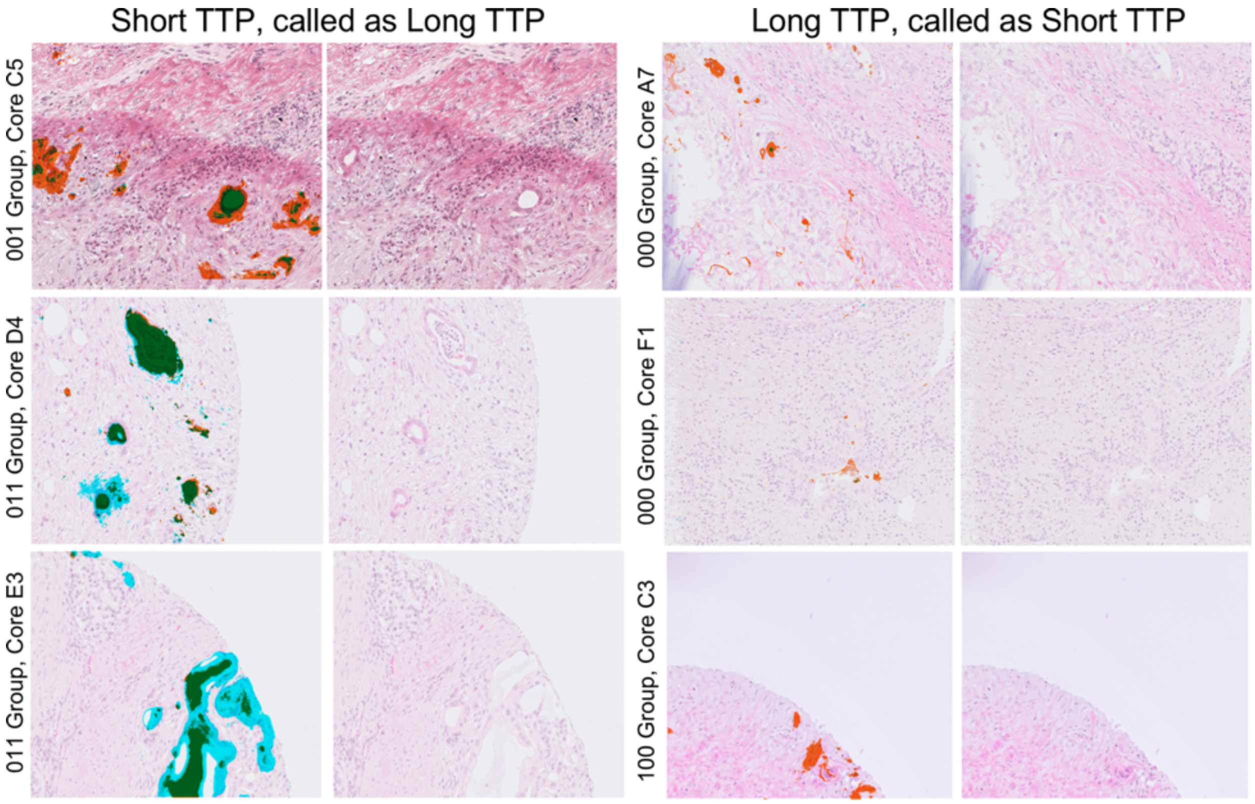

Figure S6. Images from tumors with misclassified TTP. The colors and labeling are the same as in Figure S5.
